# Maintaining a minimally invasive surgical service during a pandemic

**DOI:** 10.1007/s00383-022-05107-0

**Published:** 2022-03-25

**Authors:** Jayaram Sivaraj, Stavros Loukogeorgakis, Fiona Costigan, Stefano Giuliani, Dhanya Mullassery, Simon Blackburn, Joe Curry, Kate Cross, Paolo De Coppi

**Affiliations:** 1 Department of Specialist Neonatal and Paediatric Surgery Great, Ormond Street Hospital NHS Trust, London, UK; 2 NIHR Biomedical Research Center, Great Ormond Street Hospital, London, UK; 3 UCL GOSH Institute of Child Health, London, UK

**Keywords:** Minimally invasive surgery, Laparoscopy, COVID-19, Safety, Pandemic

## Abstract

**Purpose:**

The safety of minimally invasive surgery (MIS) was questioned in the COVID-19 pandemic due to concern regarding disease spread. We continued MIS during the pandemic with appropriate protective measures. This study aims to assess the safety of MIS compared to Open Surgery (OS) in this setting.

**Methods:**

Operations performed during 2020 lockdown were compared with operations from the same time-period in 2019 and 2021. Outcomes reviewed included all complications, respiratory complications, length of stay (LOS) and operating surgeon COVID-19 infections (OSI).

**Results:**

In 2020, MIS comprised 52% of procedures. 29% of MIS 2020 had complications (2019: 24%, 2021: 15%; *p* = 0.08) vs 47% in OS 2020 (*p* = 0.04 vs MIS). 8.5% of MIS 2020 had respiratory complications (2019: 7.7%, 2021: 6.9%; *p* = 0.9) vs 10.5% in OS 2020 (*p* = 0.8 vs MIS). Median LOS[IQR] for MIS 2020 was 2.5[6] days vs 5[23] days in OS 2020 (*p* = 0.06). In 2020, 2 patients (1.2%) were COVID-19 positive (MIS: 1, OS: 1) and there were no OSI.

**Conclusion:**

Despite extensive use of MIS during the pandemic, there was no associated increase in respiratory or other complications, and no OSI. Our study suggests that, with appropriate protective measures, MIS can be performed safely despite high levels of COVID-19 in the population.

## Introduction

Minimally Invasive Surgery (MIS) is often the operative modality of choice for most abdominal and pelvic surgery, especially due to advantages such as reduced morbidity and faster recovery times [1]. The COVID-19 pandemic has significantly impacted healthcare systems globally, and one of the early challenges was determining the safety of laparoscopic surgery [1–5]. It was hypothesised that aerosolised viral particles in the pneumoperitoneum and surgical smoke (“plume”), released when diathermy devices are used, could potentially lead to transmission of the virus [6]. This was especially concerning due to our limited pre-operative testing capacity in the early days of the pandemic [2, 4–10]. This concern was further amplified when SARS-CoV2 RNA was isolated in stool and gastrointestinal mucosa as it raised the possibility of infectious spread when entering the GI tract for surgery [3, 4, 11, 12].

Consequently, there was a lack of consensus amongst published guidelines. The surgical intercollegiate body in the UK and Ireland, suggested that open surgical approaches should be favoured over minimally invasive surgery. Others offered guidance to minimising the risk of laparoscopic surgery [2, 5, 9, 10, 13–16]. This created a significant dilemma as the evidence is compelling that MIS is more beneficial for the patient, but pandemic-related concerns meant surgeons had to consider a possibly inferior modality based on limited evidence for public health and safety of staff [17].

Our department opted to continue MIS with appropriate precautions (including personal protective equipment and suctioning of plume) as per our hospital gudelines. The aim of our study was to review whether continuing MIS with these precautions was safe for both patients and staff.

## Methods

A prospectively collected database of all MIS, Open (OS) and Endoscopic (ES) surgical procedures performed by our department during the first lockdown in England (26/03/20–15/06/20) was retrospectively reviewed. These were compared with operations from the same time period in 2019 and 2021. Demographic, clinical and SARS-CoV2 PCR test data were collected. The study was conducted with institutional audit approval (Registration No. 2867). The primary study groups of interest were MIS and OS patients. Outcomes reviewed included all postoperative complications (Clavein Dindo Score 1–5), respiratory complications (RC), length of stay (LOS) and symptomatic operating surgeon COVID-19 infections (OSI). Statistical analysis of categorical data was performed using chi-squared analysis (3 parameters) and Fischer’s Exact Test (2 parameters) while continuous data were analysed using Kruskal–Wallis test (3 parameters) and Mann–Whitney U test (2 parameters).

## Results

### Demographics

During the 2020 Lockdown between 26/03/2020 and 15/06/2020, 157 operations were performed. In comparison, 234 operations were performed in 2019 and 320 operations were performed in 2021 during the same time period. MIS comprised 52% of procedures performed in our 2020 cohort compared to 33% in our 2019 cohort and 27% in our 2021 cohort (*p* < 0.0001). 57% of operations performed in 2020 were emergency operations compared to 24% in 2019 and 19% in 2021 (*p* < 0.0001). The proportion of neonates operated on increased from 11 to 19% in 2020 and then reduced to 4% in 2021 (*p *< 0.0001). The median age [IQR] of patients undergoing surgery decreased from 2.9 [8.8] years to 1.1 [6.4] years in 2020 and then increased to 3 [6.1] years in 2021 (*p* = 0.02). The median weight [IQR] followed a similar pattern and decreased from 15 kg [18.7] to 9.7 kg [17.4] in 2020 and subsequently rose to 15.1 kg [17.7] in 2021 (*p* = 0.002) in concordance with this (Table1).

**Table 1:** Demographics of patients undergoing surgery

	2019	2020	2021	*P* Value
Total patient number	234	157	320	NA
MIS (%)	78 (33%)	82 (52%)	87 (27%)	< 0.0001
OS (%)	122 (52%)	66 (42%)	168 (53%)	0.07
ES (%)	34 (15%)	9 (6%)	64 (20%)	0.0002
Emergency (%)	55 (24%)	89 (57%)	60 (19%)	< 0.0001
Neonates (%)	25 (11%)	29 (19%)	14 (4%)	< 0.0001
Male (%)	111 (47%)	106 (68%)	188 (58%)	0.0003
Median age (years; IQR)	2.9 (8.8)	1.1 (6.4)	3 (6.1)	0.02
Median weight (kg; IQR)	15 (18.7)	9.7 (17.4)	15.1 (17.7)	0.002

The proportion of MIS that was emergency surgery increased from 28% in 2019 to 50% in 2020 and subsequently decreased to 22% in 2021 (*p* = 0.003). Similarly, the proportion of OS that was emergency surgery increased from 22% in 2019 to 70% in 2020 and then subsequently decreased to 21% in 2021 (*p* < 0.0001; Table 2).

**Table 2:** Patients undergoing emergency surgery

	2019	2020	2021	*P* value
Emergency MIS (% of total MIS)	22 (28%)	41 (50%)	19 (22%)	0.0003
Emergency OS (% of total OS)	27 (22%)	46 (70%)	35 (21%)	< 0.0001
Emergency ES (% of total ES)	6 (17%)	2 (22%)	6 (9%)	0.34

The proportion of OS that was neonatal surgery increased from 14 to 32% between 2019 and 2020 and subsequently reduced to 7% in 2021 (*p* < 0.0001) while there was no significant change in neonatal MIS or ES (Table 3).

**Table 3:** Neonatal patients undergoing surgery

	2019	2020	2021	*P* value
Neonatal MIS (% of total MIS)	8 (10%)	8 (10%)	2 (2%)	0.08
Neonatal OS (% of total OS)	17 (14%)	21 (32%)	12 (7%)	< 0.0001
Neonatal ES (% of total ES)	0 (0%)	0 (0%)	0 (0%)	1

**Fig. 1:**
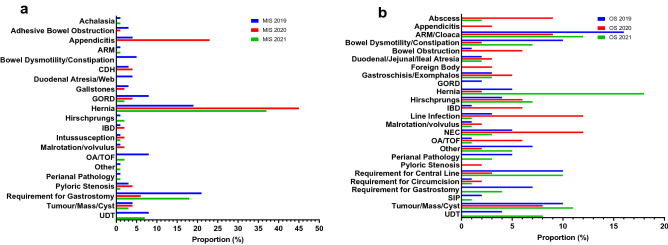
**a** Indications for minimally invasive surgery. **b** Indications for open surgery

The indications for MIS were noticeably different in 2020. The majority of patients in 2020 required MIS for inguinal herniae or appendicitis, whereas the indications in 2019 were far more varied. In 2021, hernia was the most common indication for surgery, but different procedures had also resumed. (Fig. 1a).

The most common indication for OS was ARMs/Cloaca in 2019; however, this was reduced dramatically in 2020. This increased in 2021 but was not back at 2019 levels. The most common indications for OS in 2020 were Line infection, NEC, ARM/Cloaca and Abscesses. The latter was not an indication for surgery in 2019. In 2021, herniae were the most common indication for OS, and surgery for UDT had resumed following no operations for this in 2020 (Fig. 1b).

### Length of stay

**Fig. 2:**
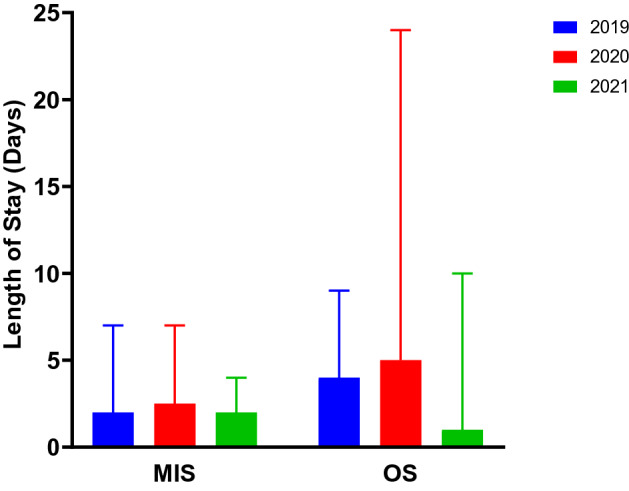
Length of stay

Median LOS [IQR] for MIS was 2.5 [6] days in 2020. In 2019, this was 2 [5.8] days, and in 2021, this was 2 [3] days (*p* = 0.8 vs 2020). Meanwhile, OS patients had a median LOS of 5 [18] days in 2020. In 2019, this was 4 [9] days and in 2021 this was 1 [9] days (*p* = 0.2 vs 2020). There was no significant difference in LOS between MIS and OS in 2019 (*p* = 0.6), 2020 (*p* = 0.06) or 2021 (*p* = 0.5) (Fig. 2).

### Complications

**Fig. 3:**
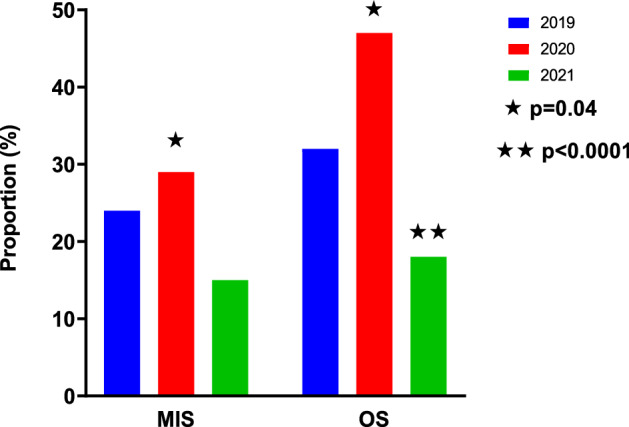
All post-operative complications

In 2020, 29% of MIS patients had a post-operative complication compared to 24% in 2019 and 15% in 2021 (*p* = 0.08 vs 2020). In 2020, 47% of OS patients had a post-operative complication compared to 32% in 2019 and 18% in 2021 (*p* < 0.0001 vs 2020). There was no significant difference in complications between MIS and OS in 2019 (*p* = 0.3) or 2021 (*p* = 0.6) but there was a significant difference in 2020 (*p* = 0.04; Fig. 3).

**Fig. 4:**
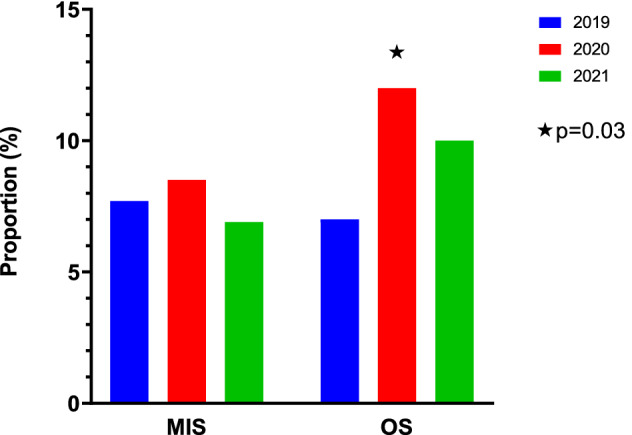
Post-operative respiratory complications

In 2020, 8.5% of MIS patients had a post-operative RC compared to 7.7% in 2019 and 6.9% in 2021 (*p* = 0.9 vs 2020). In 2020, 12.1% of OS patients had a post-operative RC compared to 6.9% in 2019 and 10.5% in 2021 (*p* = 0.03 vs 2020). There was no significant difference in RC between MIS and OS for all three years (2019 *p* = 1; 2020 *p* = 0.8; 2021 *p* = 0.6). Post-operative PCR testing for COVID-19 was not routinely performed. Only 4.9% of MIS patients and 7.% of OS patients were tested. None of these patients tested positive (Fig. 4).

### COVID-19 infection and safety

There was only 1 COVID-19 Positive patient undergoing MIS and OS respectively during our study period. Both of these patients underwent surgery during the 2020 lockdown. None of the operating surgeons for those two procedures developed COVID-19 symptoms (Table 4).

**Table 4:** Operating surgeon infection

	MIS		OS	
	2020	2021	2020	2021
COVID-19 positive patient (%)	1 (1.2%)	0 (0%)	1 (1.5%)	0 (0%)
Surgeons with COVID-19 symptoms (%)	0 (0%)	0 (0%)	0 (0%)	0 (0%)

## Discussion

Our study describes the change in practice at our centre over the course of the COVID-19 pandemic. This includes first lockdown in 2020 compared to the same time in 2019 prior to the pandemic as well as in 2021—an entire year after the initial onset of the pandemic.

While we were able to maintain MIS during the pandemic, we have seen interesting transitions in our practice, including our patient demographics during our study period. During the initial 2020 lockdown, the total number of operations performed reduced dramatically from 234 procedures in 2019 to 157 in 2020. However in 2021, we surpassed 2019 operation numbers with 320 procedures performed in the same time period. This phenomenon is likely to be secondary to the collapse of elective operating in 2020 combined with increased conservative management of surgical pathology [19, 20]. In addition, there was an accelerated effort in 2021 to resume elective services to deal with the large backlog from 2020 and this was facilitated by having a larger body of consultant surgeon﻿s.

Despite the reduction in operations, the proportion of patients undergoing MIS increased significantly (*p* = 0.0002) and formed the majority of operations performed in 2020 (57%; Table 1). This interesting turn of events, despite the initial fear around MIS, is likely due to the increased number of inguinal hernia repairs and appendicectomies performed at our institution during the pandemic (Fig. 1a). It is important to note that it is unlikely that there was actually an increase in patients presenting with these conditions. Due to the increased demand on adult services and reduced paediatric operating capacity at hospitals within the North Central London region, paediatric surgical services were centralised at our institute. Therefore, patients who may have had appendicectomies performed by adult surgeons or hernia repairs performed at other paediatric surgical centres were transferred to our care. Our 2021 data corroborates this as no appendicectomies were performed during the 2021 study period once neighbouring hospitals resumed their paediatric surgical services. We note that hernia repairs continued to be a common indication for MIS in 2021 and this was likely to reflect the need to continue performing elective repairs.

It is well known that appendicitis and inguinal hernias are two of the most common indications for emergency surgery in the paediatric population, and laparoscopy is often the preferred approach for these conditions in our institution (particularly for neonates with herniae) due to the advantages it confers [20–22]. We can see that the impact of the pandemic on elective services was significant, as the proportion of Emergency Surgery performed transitioned from 24% in 2019 to 57% in 2020 *p* < 0.0001. The effort made to resume elective services and clear the surgical backlog appears to have dramatically dropped the proportion of emergency surgery down to 19% in 2021 (*p* < 0.0001).

Likewise, we saw that OS patients had different indications for surgery in 2020. Open hernia repairs continued to be performed throughout the three years but there was a sharp jump in 2021 to 11% (4% in 2019; 3% in 2020), reflecting the attempt to clear elective backlogs caused by the pandemic. Abscesses were not an indication for surgery in 2019 but 14% of patients undergoing OS in 2020 had abscesses as we took on operative work from neighbouring centres during the pandemic's peak. In 2021, this was only 1% and reflects the transition back to normal practice. Similarly, no surgery was undertaken for Undescended Testes (UDT) in 2020 as elective surgeries were postponed and 2021 levels were higher than 2019, yet again reflecting the attempt to clear elective backlogs. Interestingly, we found that there was a marked increase in surgery for line infections in 2020. There have been reports of increased rates of line infection during the pandemic in the literature; however, this appears to be primarily be related to more adults requiring treatment in intensive care [18, 23]. We suspect that increased surgery for line infections at our institute was more likely to once again be related to us taking on additional emergency work from neighbouring hospitals.

Other demographic changes that we noted to be significant included the increase in percentage of neonatal procedures and male patients (neonates *p* < 0.0001 vs 2020; male *p* = 0.0003 vs 2020) while patient age and weight decreased. The increase in neonatal procedures is likely secondary to the increased focus on emergency surgery. We noted that the percentage of OS that was emergency surgery increased significantly to 70% in 2020 (22% in 2019, 21% in 2021; *p* < 0.0001 vs 2020) and that the percentage of OS that involving neonates increased along with this. Figure 1a and b also show that common neonatal pathologies such as NEC, Intestinal Atresia and Gastroschisis/Exomphalos formed a larger proportion of total operating in 2020 for both OS and MIS.

The significant decrease in median weight and age of the patients undergoing surgery in 2020 is also explained by the increase in neonatal surgery (N.B. there is no substantial evidence that correlates COVID-19 with prematurity or low birthweight) [24]. The increase in hernia repairs described earlier explains the increase in males requiring surgery due to their increased risk of developing inguinal herniae [25].

There was no significant difference in complications for MIS across the three years (*p* = 0.08). Interestingly, this was not the case for the OS group. The 2020 OS group had a significantly higher complication rate compared to their 2019 and 2021 counterparts (*p* < 0.0001 vs 2020). Furthermore, there was a significant difference between MIS and OS complication rates in 2020 (29% vs 47%; *p* = 0.04) but this was not the case in 2019 and 2021 (Fig. 3). This suggests that postoperative outcomes were better in our patients undergoing MIS and that patients undergoing OS appeared to have the least favourable outcomes. Given that these patients were primarily undergoing emergency surgery and many were neonates who are more likely to develop complications, these findings are unsurprising [26]. These patients are more likely to be premature and have multiple comorbidities which not only increases the anaesthetic risk but also predisposes them to further complications and conversion to OS [26]. Furthermore, there was an increase in proportion of emergency surgery, which inherently by nature are higher risk than elective.

When specifically reviewing respiratory complications which could be associated with viral infections including SARS-CoV2, there was no significant difference for the MIS group across all three years. (8.5% in 2020, 7.7% in 2019, 6.9% in 2021; *p* = 0.9 vs 2020). However, there was significant difference in the OS group (12.1% in 2020, 6.9% in 2019, 10.5% in 2021; *p* = 0.03 vs 2020). This finding is likely to once again be a reflection of the changing demographics of our patients (Tables 1, 2, 3 & Fig. 2). With less elective surgery and more complex neonates undergoing emergency OS within this group, it is understandable that these patients were more likely to develop respiratory complications (especially due to the presence of comorbidities such as RDS) [26].

We see a similar pattern with LOS data where the increase in median LOS was minimal and not statistically significant for MIS in 2020 (2.5 [6] days in 2020 vs 2 [5.8] days in 2019 and 2 [3] days in 20,201; *p* = 0.8). There was a slightly larger increase in median LOS for 2020 OS patients (5 [23] days in 2020 vs 4 [9] days in 2019 and 1[9] days in 2021). This was likely caused by the increase in emergency operating in complex neonates in 2020 compared to the higher volume of elective surgery in 2019 and particularly in 2021. However, this difference was found to not be statistically significant (*p* = 0.2). Furthermore, when comparing median LOS between the two operative modalities in each year, we once again found no significant difference (*p* = 0.6 in 2019, *p* = 0.06 in 2020, *p* = 0.5 in 2021). Thus, our data suggest that in spite of the increased OS complication rate in 2020, the median LOS for our patients was not significantly impacted.

Finally, our data on safety and the spread of COVID-19 infection in surgeons was limited by low number of COVID-19 positive patients undergoing surgery at our centre. This is probably because of the low incidence of COVID-19 infection in children [27]. However, our limited data do suggest that the risk to operating surgeons is low when taking appropriate precautions as no surgeon developed symptomatic infections postoperatively.

It is important to note that several viruses such as HBV, HPV and HIV have previously been found in surgical smoke but the infectivity of these aerosolised viral particles is unclear [1–3, 7–9, 28]. While SARS-CoV2 has not yet been identified in surgical smoke, there was initially no evidence to rule out the alarming theoretical risk of spreading COVID-19 following MIS. However, one could argue MIS might be safer as the release of pneumoperitoneum/pneumothorax and surgical smoke could be controlled and carefully suctioned out at the end of a procedure, whereas the spread of the plume in open surgery cannot be controlled so easily [8, 29]. Furthermore, the advantages of reduced morbidity and faster recovery were difficult to ignore in the context of a pandemic where health services across the world have been under immense strain and risked being overwhelmed by demand.

Therefore, despite the limitations of our study and the lack of data regarding spread of SARS-CoV2 viral particles in surgical smoke, we feel it is safe to conclude that MIS was not only safe for patients and surgeons, but also offers considerable advantages with regards to surgical morbidity.

## Conclusion

There was a significant change in the demographics of patients undergoing surgery in our department during the first wave of COVID-19. In 2020, there was reduced overall operating with increased use of MIS and a significant skew towards emergency surgery. Despite the initial concern surrounding MIS, we did not see an increase in LOS or complications (respiratory or otherwise) in the 2020 MIS group. Surgeons operating on COVID-19 positive patients did not develop any symptoms when appropriate protective measures were taken. In conclusion, our data suggest that with appropriate precautions (including PPE), MIS during the COVID-19 pandemic was safe for both patients and surgeons.

## References

[1] Angioni S (2020). Laparoscopy in the coronavirus disease 2019 (COVID-19) era. Gynecol Surg.

[2] Francis N, Dort J, Cho E (2020). SAGES and EAES recommendations for minimally invasive surgery during COVID-19 pandemic. Surg Endosc.

[3] Vigneswaran Y, Prachand VN, Posner MC (2020). What is the appropriate use of laparoscopy over open procedures in the current COVID-19 climate?. J Gastrointest Surg.

[4] Ribeiro SC, Lauletta ALF, Franco BC (2020). Laparoscopic surgery and coronavirus disease: What do we know now?. Clinics (Sao Paulo, Brazil).

[5] el Boghdady M, Ewalds-kvist BM (2020). Laparoscopic Surgery and the debate on its safety during COVID-19 pandemic: A systematic review of recommendations. The Surgeon.

[6] Pavan N, Crestani A, Abrate A (2020). Risk of virus contamination through surgical smoke during minimally invasive surgery: a systematic review of the literature on a neglected issue revived in the COVID-19 pandemic era. Eur Urol Focus.

[7] Veziant J, Bourdel N, Slim K (2020). Risks of viral contamination in healthcare professionals during laparoscopy in the Covid-19 pandemic. J Visc Surg.

[8] Mintz Y, Arezzo A, Boni L (2020). The risk of COVID-19 transmission by laparoscopic smoke may be lower than for laparotomy: a narrative review. Surg Endosc.

[9] Prato AP, Conforti A, Almstrom M (2020). Management of COVID-19-positive pediatric patients undergoing minimally invasive surgical procedures: Systematic review and recommendations of the board of European society of pediatric endoscopic surgeons. Front Pediatr.

[10] di Saverio S, Khan M, Pata F (2020). Laparoscopy at all costs? Not now during COVID-19 outbreak and not for acute care surgery and emergency colorectal surgery: A practical algorithm from a hub tertiary teaching hospital in Northern Lombardy, Italy. J Trauma Acute Care Surg.

[11] Yang L, Tu L (2020). Implications of gastrointestinal manifestations of COVID-19. Lancet Gastroenterol Hepatol.

[12] Pavan N, Crestani A, Abrate A, De Nunzio C, Esperto F, Giannarini G, Galfano A, Gregori A, Liguori G, Bartoletti R, Porpiglia F, Simonato A, Trombetta C, Tubaro A, Ficarra V, Novara G, Research Urology Network (RUN) (2020). Risk of virus contamination through surgical smoke during minimally invasive surgery: a systematic review of the literature on a neglected issue revived in the COVID-19 pandemic era. Eur Urol Focus..

[13] British Association of Paediatric Endoscopic Surgeons. Bapes.org.uk. 2022. Available from: https://www.bapes.org.uk/s/BAPES-COVID19-2203.pdf.

[14] De Leeuw RA, Burger NB, Ceccaroni M, Zhang J, Tuynman J, Mabrouk M, Barri Soldevila P, Bonjer HJ, Ankum P, Huirne J (2020). COVID-19 and laparoscopic surgery: scoping review of current literature and local expertise. JMIR Public Health Surveill..

[15] Intercollegiate General Surgery Guidance on COVID-19 UPDATE | RCSEd [Internet]. The Royal College of Surgeons of Edinburgh. 2022. Available from: https://www.rcsed.ac.uk/news-public-affairs/news/2020/march/intercollegiate-general-surgery-guidance-on-covid-19-update.

[16] Laparoscopy in The Covid-19 Environment – ALSGBI Position Statement - ALSGBI [Internet]. ALSGBI - The UK & Ireland’s Premier Professional Association in the field of Laparoscopic Surgery. 2022 Available from: https://www.alsgbi.org/2020/04/22/laparoscopy-in-the-covid-19-environment-alsgbi-position-statement/.

[17] Hadjittofi C, Seraj SS, Uddin A (2021). Laparoscopic vs open surgery during the COVID-19 pandemic: what are the risks?. Ann R Coll Surg Engl.

[18] Patel PR, Weiner-Lastinger LM, Dudeck MA (2021). Impact of COVID-19 pandemic on central-line–associated bloodstream infections during the early months of 2020, National Healthcare Safety Network. Infect Control Hosp Epidemiol.

[19] Clements JM, Burke JR, Hope C (2021). The quantitative impact of COVID-19 on surgical training in the United Kingdom. BJS Open.

[20] Bethell GS, Rees CM, Sutcliffe JR, Hall NJ (2020). Management and early outcomes of children with appendicitis in the UK and Ireland during the COVID-19 pandemic: A survey of surgeons and observational study. BMJ Paediatr Open.

[21] Ho IG, Ihn K, Koo EJ (2018). Laparoscopic repair of inguinal hernia in infants: Comparison with open hernia repair. J Pediatr Surg.

[22] Nah SA, Giacomello L, Eaton S (2011). Surgical repair of incarcerated inguinal hernia in children: Laparoscopic or open?. Eur J Pediatr Surg.

[23] Fakih MG, Bufalino A, Sturm L (2021). Coronavirus disease 2019 (COVID-19) pandemic, central-line–associated bloodstream infection (CLABSI), and catheter-associated urinary tract infection (CAUTI): The urgent need to refocus on hardwiring prevention efforts. Infect Control Hosp Epidemiol.

[24] de Melo GC, de Araújo KCGM (2020). COVID-19 infection in pregnant women, preterm delivery, birth weight, and vertical transmission: a systematic review and meta-analysis. Cadernis de Saude Publica.

[25] Chang SJ, Chen JYC, Hsu CK (2015). The incidence of inguinal hernia and associated risk factors of incarceration in pediatric inguinal hernia: a nation-wide longitudinal population-based study. Hernia.

[26] Platt MJ (2014). Outcomes in preterm infants. Public Health.

[27] Mehta NS, Mytton OT, Mullins EWS (2020). SARS-CoV-2 (COVID-19): What do we know about children? a systematic review. Clin Infect Dis.

[28] Kwak HD, Kim SH, Seo YS, Song KJ (2016). Detecting hepatitis B virus in surgical smoke emitted during laparoscopic surgery. Occup Environ Med.

[29] Agrawal V, Sharma D (2020). Initial advice to avoid Laparoscopic Surgery due to fear of COVID-19 virus transmission: Where was the evidence?. Br J Surg.

